# Impact of a Peer-Led International Training Program on Work Motivation Among Early-Career Psychiatrists: A Mixed-Methods Study

**DOI:** 10.7759/cureus.93012

**Published:** 2025-09-23

**Authors:** Toshihiro Shimizu, Junko Kitaoka, Ken Suzutani, Yuto Satake, Masahide Koda, Izumi Kuramochi, Norman Sartorius

**Affiliations:** 1 Department of Psychiatry, Saitama Prefectural Psychiatric Hospital, Ina, JPN; 2 Department of Psychiatry, Fukkoukai Tarumi Hospital, Kobe, JPN; 3 Department of Psychiatry, Aizu Medical Center, Aizuwakamatsu, JPN; 4 Department of Psychiatry, The University of Osaka, Suita, JPN; 5 Co-learning Community Healthcare Re-innovation Office, Graduate School of Medicine, Okayama University, Okayama, JPN; 6 Department of Epileptology and Psychiatry, National Center of Neurology and Psychiatry, Kodaira, JPN; 7 Psychiatry, Association for the Improvement of Mental Health Programs (AIMHP), Geneva, CHE

**Keywords:** cadp, early-career psychiatrists, jypo, peer-led training, peer networking, professional development, professional identity, work motivation

## Abstract

Background

The Japan Young Psychiatrists Organization (JYPO) has conducted a Course for Academic Development of Psychiatrists (CADP), a peer-led residential international training program, since 2002 to promote the professional development of early-career psychiatrists. This study aimed to evaluate the impact of CADP on participants' work motivation using a psychometric scale and to identify the factors contributing to these changes.

Methods

We conducted a mixed-method study with 23 Japanese participants of the 21st CADP from March 8 to 10, 2024, in Himeji, Japan. Work motivation was assessed using the abbreviated version of the Measure of Multifaceted Work Motivations (MWM-12) at two time points: two weeks before and three months after the course. The total and subitem scores of the MWM-12 were analyzed using the Wilcoxon signed-rank test. Furthermore, free-text responses collected before and after the course were subjected to qualitative analyses.

Results

Significant improvements were observed in the MWM-12 total score from pre-course to post-course. Significant increases were also identified in specific sub-items: M1 (directionality of achievement-oriented motivation), M4 (directionality of competition-oriented motivation), M6 (sustainability of competition-oriented motivation), and M9 (sustainability of cooperation-oriented motivation). Qualitative analysis revealed changes in key categories, including growth as a psychiatrist, personal networking, personal growth, and increased motivation. The integration of quantitative and qualitative findings suggested that enhanced career perspectives (M1), professional growth and peer interaction (M4), and increased self-confidence and support networks (M6 and M9) contributed to improved motivation.

Conclusion

This study demonstrated that a three-day, two-night peer-led training program positively influenced work motivation among early-career psychiatrists.

## Introduction

Work motivation is defined as a psychological process that orients, activates, and sustains behavior toward achieving a goal [1]. It is conceptualized in terms of three dimensions: direction, intensity, and sustainability. Among healthcare workers, motivation reflects the dynamic interaction between individuals and their work environment, which can considerably influence the quality of healthcare services [2].

Recently, increasing attention has been directed toward improving the work-life balance of healthcare providers, including clinicians and staff. Indeed, the "Triple Aim" framework in healthcare systems, enhancing patient experience, improving population health, and reducing healthcare costs, has evolved into the "Quadruple Aim" through the addition of a fourth goal: improving the work-life of healthcare professionals [3]. Studies have indicated that healthcare providers with high levels of intrinsic motivation are more likely to exhibit positive behaviors and attitudes in their interactions with patients [4]. A study of early-career psychiatrists in Japan found that high occupational satisfaction, likely linked to higher levels of work motivation, was associated with interest in work content, autonomy, opportunities for growth and career development, and ease of communication with supervisors and colleagues [5].

Conversely, residents at high risk for burnout or depression have been reported to commit more medical errors and are less likely to adhere to established best-practice principles in non-psychiatric specialties [6]. Psychiatrists may be particularly vulnerable to burnout owing to profession-specific stressors, including patient violence, limited resources, and professional isolation, which can undermine motivation and contribute to burnout [7]. A nationwide study in Japan, which received responses from 704 psychiatrists, reported that 148 (21%) of psychiatrists experienced high levels of emotional exhaustion, which is recognized as a core element of burnout. Three hundred and eleven (46%) respondents indicated difficulties in achieving work-life balance, and 507 (72%) reported low personal accomplishment, a proportion higher than the corresponding rates in other countries [8]. Specifically, data from the Japanese cohort of Burnout Syndrome Among Psychiatric Trainees (BoSS International) study indicated that, in line with international results, severe burnout among early-career psychiatrists was significantly correlated with long working hours, limited supervisory consultations, and a lack of regular breaks [9].

The factors influencing the work motivation of healthcare professionals can be categorized into intrinsic and organizational factors. According to the self-determination theory, intrinsic motivation stems from the satisfaction of three fundamental psychological needs: autonomy, competence, and relatedness [10]. Autonomy is closely linked to an individual's professional identity and career vision, as these elements reflect the goals and values that guide their professional trajectory. Competence is associated with self-efficacy, which represents the belief in one's capability to effectively perform tasks. Relatedness pertains to the quality of interpersonal relationships within the workplace and connections within professional networks, fostering a sense of belonging and mutual support.

Organizational factors influencing healthcare professionals' motivation can be categorized into financial and nonfinancial aspects. Financial factors include salary, allowances, and performance-based compensation. In contrast, non-financial factors include professional growth and training opportunities, promotion and career advancement, a positive work environment, and effective management and leadership [11]. Nonfinancial factors such as opportunities for personal development, career advancement, and collaborative workplace relationships have been reported to be crucial in enhancing work motivation [12].

For healthcare providers, the formation of professional identity is an essential process for maximizing professional competence, as it influences personal growth, self-efficacy, and work motivation. Enhancing professionalism during the early stages of one's career (for example, by reaffirming ethical principles, patient-centeredness, and cultivating a sense of mission as a physician) is thought to prevent motivational decline and promote resilience in the face of challenges [13]. This process is dynamic and evolves across multiple stages of professional education, clinical experience, and interpersonal interactions [14].

Psychiatrists need opportunities to cultivate multifaceted competencies during early career training, as their role encompasses not only understanding and addressing patients' distress in an integrated manner as clinicians, but also guiding patients and their families within the healthcare system and engaging in public health initiatives [15]. To support this development, structured curricula for early-career psychiatrists have been developed and implemented in various regions worldwide, aiming to provide essential skills for scientific presentation, communication, and leadership [16]. Over the past two decades, approximately 3,000 individuals have participated in such programs, which have served as turning points for many professional careers [17].

As part of this global effort, the Course for Academic Development of Psychiatrists (CADP) was launched in 2002 as a training program designed to enhance academic and leadership skills and foster professional networking among early-career psychiatrists. The course includes various sessions, such as oral and poster presentations, special lectures, and group work. All activities are conducted entirely in English, which is the official language throughout the program. Annually organized by the Japan Young Psychiatrists Organization (JYPO), the CADP has welcomed participants from both Japan and abroad [18]. The steering committee, composed of early‑career psychiatrists, ensured that CADP met the needs of its participants-reflecting a peer‑led structure. CADP is acknowledged as a valuable Leadership and Professional Skills Course for early-career psychiatrists, a perception shared internationally [19]. Even during the COVID-19 pandemic, CADP was not postponed; instead, a JYPO Online International Networking (JOIN) meeting was held to ensure that early-career psychiatrists could continue to develop academic skills and maintain their professional connections [20,21]. Over the 20 years since its inception, CADP has helped to produce graduates who have become key figures in universities, research institutions, and clinical settings. Several alumni of CADP have reported that participation in the program positively influenced their career development [22,23]. These outcomes suggest that participation in CADP may enhance work motivation. However, even if the motivation to work increases, the effect may be temporary.

Therefore, this study aimed to examine changes in the work motivation of participants in the 21st CADP, measured from two weeks before to three months after the course, and to identify the factors contributing to these changes. Furthermore, this study sought to offer insights into the key components that may enhance the design of future training programs for early-career psychiatrists, making them more appealing and motivating for participants aspiring to become competent professionals.

## Materials and methods

Study design

This study employed a mixed-method approach with a convergent design to investigate changes in work motivation and the factors contributing to these changes among participants in the 21st CADP. A quantitative approach was used to evaluate the sustained effects of participation on work motivation, specifically focusing on changes persisting for three months post-course. Concurrently, a qualitative approach explored the types of changes participants experienced through their participation in the CADP. Finally, the quantitative and qualitative findings were integrated to address a third research question regarding the factors that contribute to changes in work motivation following participation in CADP.

**Figure 1:**
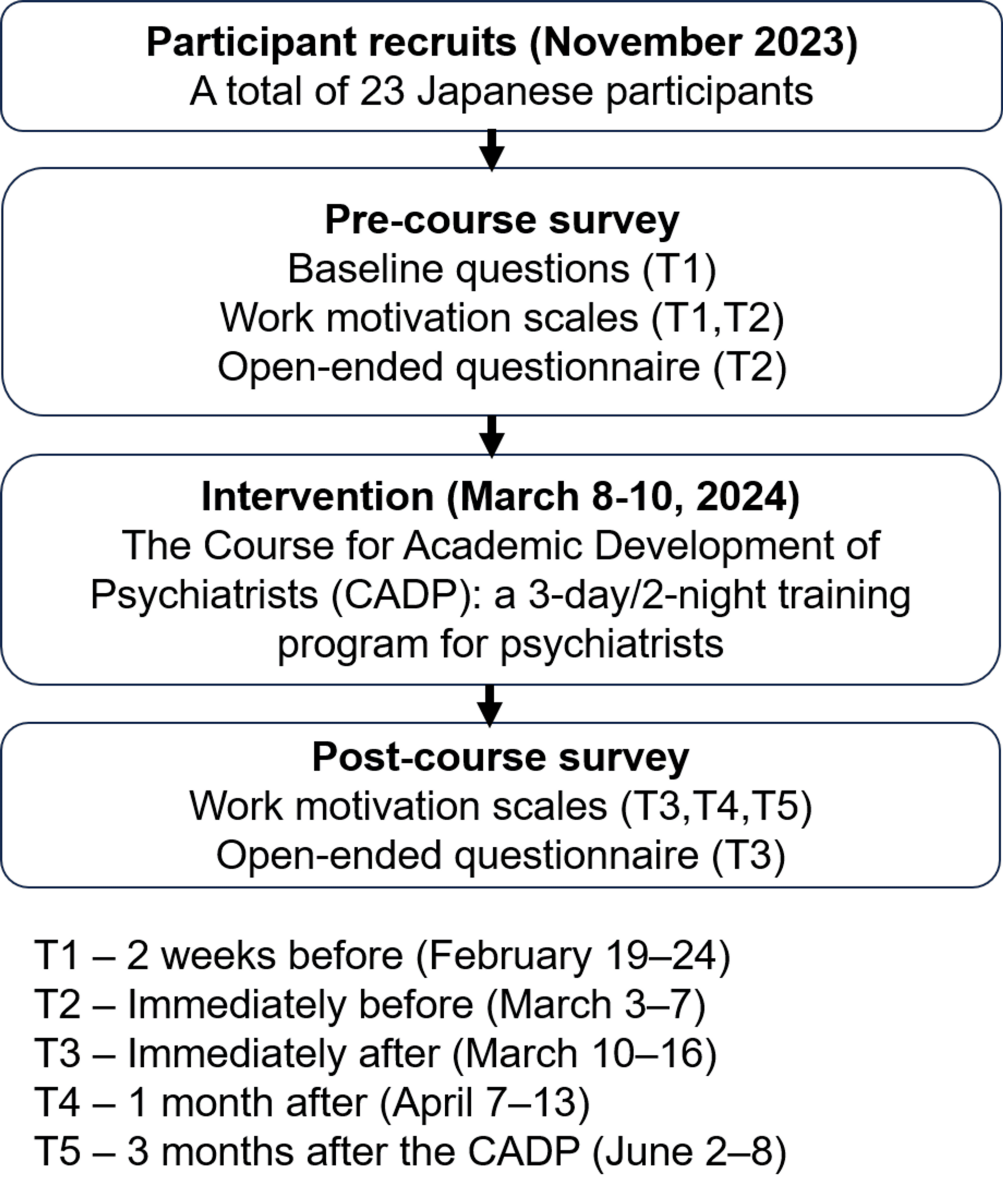
Structure of the online questionnaire survey (T1–T5) This figure illustrates the content and structure of the online questionnaire administered at five time points (T1–T5).

The online survey included both the abbreviated version of the Measure of Multifaceted Work Motivations (MWM-12) and open-ended questions developed by the authors. The survey was distributed to the participants of the 21st CADP at five time points (Figure 1). These survey periods, each lasting one week, were as follows: T1 (two weeks before, February 19-24), T2 (immediately before, March 3-7), T3 (immediately after, March 10-16), T4 (one month after, April 7-13), and T5 (three months after the CADP, June 2-8). The survey employed a within-subject design with five repeated measurements, using the first two as a baseline to evaluate changes following the intervention. This approach allowed each participant to serve as their own control, enabling a more precise evaluation of the changes brought about by the intervention. This design has the advantage of controlling for potential confounding factors that are unique to each individual, thereby minimizing individual variability.

Nonrespondents received reminders via email or messaging applications during each response window. Participation was voluntary, and informed consent was obtained from all participants prior to their inclusion in the study. Participants were informed that certain members of the 21st CADP Steering Committee were conducting the research. To ensure confidentiality and prevent personal identification, names were collected solely to track responses and were subsequently anonymized during data analysis. The Ethical Review Committee of the Fukkoukai Tarumi Hospital issued approval number T23-04.

The 21st CADP

The 21st CADP was held from March 8 to 10, 2024, in Himeji, Japan (Days 1 and 2: 8:30-21:30; Day 3: 8:30-13:00). The course comprised oral and poster presentations, lectures on presentation skills, special lectures by invited experts, workshops, and social events, including reception gatherings. The course was designed to maximize participant involvement, providing opportunities for individual presentations, comment sessions during peer presentations, and continuous involvement in group work and other activities. All sessions were organized by JYPO secretariat staff and members of the CADP Steering Committee (eight of whom also participated in the course). The Steering Committee comprised volunteers who had previously participated in CADP. As participants graduated after attending CADP four times, the committee was consistently composed of early-career psychiatrists. Appendix 1 provides an overview of the program’s content and links to reports from previous courses.

Participants

All Japanese participants in the 21st CADP were recruited for this study. The call for participation was disseminated through domestic and international networks of early-career psychiatrists and made publicly available on the official JYPO website. The application criteria were as follows: applicants were required to have no more than 12 years of mental health experience, full attendance for the entire course was mandatory, and in-person attendance was required without an option for online participation.

​​​​​​A total of 28 participants were selected, including 23 from Japan, one from India, two from Malaysia, one from Singapore, and one from Thailand.

Measurements

The Measure of Multifaceted Work Motivations (MWM) is a validated scale that assesses work motivation across three dimensions, (i) directionality, (ii) intensity, and (iii) sustainability, and four types of motivation (i) achievement-oriented (drive to complete assigned tasks), (ii) competition-oriented (desire to outperform colleagues), (iii) cooperation-oriented (willingness to collaborate with colleagues), and (iv) learning-oriented (autonomous pursuit of knowledge and skill development) [24].

In this study, the abbreviated version of the MWM, referred to as MWM-12, was used. This 12-item scale is categorized across the same three dimensions and four motivational orientations as the full MWM. The scale includes three items each for achievement (M1-M3), competition (M4-M6), cooperation (M7-M9), and learning (M10-M12).

Each item was rated on a 5-point Likert scale ranging from 1 (strongly disagree) to 5 (strongly agree). The MWM-12 yields a total score ranging from 12 to 60, with higher scores indicating greater overall work motivation.

Participants were also asked to respond to open-ended questions at T2 and T3. At T2 (immediately preceding the course), they described their expectations ("What are your expectations for the CADP?") and their purposes for participating ("What is your purpose for participating in the CADP?"). At T3 (immediately following the course), participants reflected on their experiences by responding to the following prompts: perceived gains ("What did you benefit from your participation in the CADP?"), findings ("What is new or surprising to you after participating in the CADP?"), and general impressions ("Please write your impressions of your participation in the CADP.").

Analyses

Changes in the mean total MWM-12 score were described, and transitions across the five assessment points were visualized using Python. OpenAI’s GPT-4o (OpenAI, Inc., San Francisco, CA, USA) assisted with code generation, and all scripts were reviewed and refined by the authors. To assess the sustained effects of CADP participation, changes in the total and sub-item scores between T1 and T5 were analyzed using the Wilcoxon signed-rank test. All statistical analyses were performed using SPSS Statistics for Windows, Version 25 (IBM Corp., Armonk, NY, USA). Statistical significance was defined as a two-tailed *p*-value < 0.05.

Participants’ free-text responses were analyzed using inductive content analysis, guided by general principles of grounded theory such as open coding. These responses included pre- (expectations and participation objectives) and post-course (perceived gains, findings, and general impressions) data. For the initial coding stage, TS and JK independently and repeatedly read the transcripts to familiarize themselves with the data, and performed the initial stage of coding. TS then manually developed the first draft of categories. Separately, MK used the AI tool Claude 3.5 Opus (Anthropic, San Francisco, CA, USA) to support the open coding process. The prompt "Analyze the following list of responses based on grounded theory" was used to generate an initial set of codes and categories. MK critically reviewed the AI-generated output and made independent judgments when finalizing this second draft of categories. After both drafts were completed, the research team (TS, JK, KS, YS, and IK) held four discussion sessions. During these meetings, all members collaboratively re-read the transcripts and compared the two drafts to refine the categorization. Through these discussions, final categories and subcategories were determined, and the resulting codebook, which defines each category and subcategory, is provided in Appendix 2.

No pilot study was conducted before this investigation. Study validity was confirmed by applying the Consolidated Criteria for Reporting Qualitative Research, a 32-item checklist designed for interviews and focus groups [25].

## Results

Demographics

The study sample comprised 23 Japanese participants. The mean age of the participants was 32.9 years. There were 19 male (82%) and four female (18%) participants. Membership in the JYPO was reported by 17 participants (74%), and the mean duration of psychiatric practice within the sample was 4.0 years. The cohort included one junior resident prior to psychiatric specialization and two medical students. Twelve participants (52%) were first-time attendees of the CADP. Comprehensive participant profiles are presented in Appendix 3.

Work motivation change

**Figure 2:**
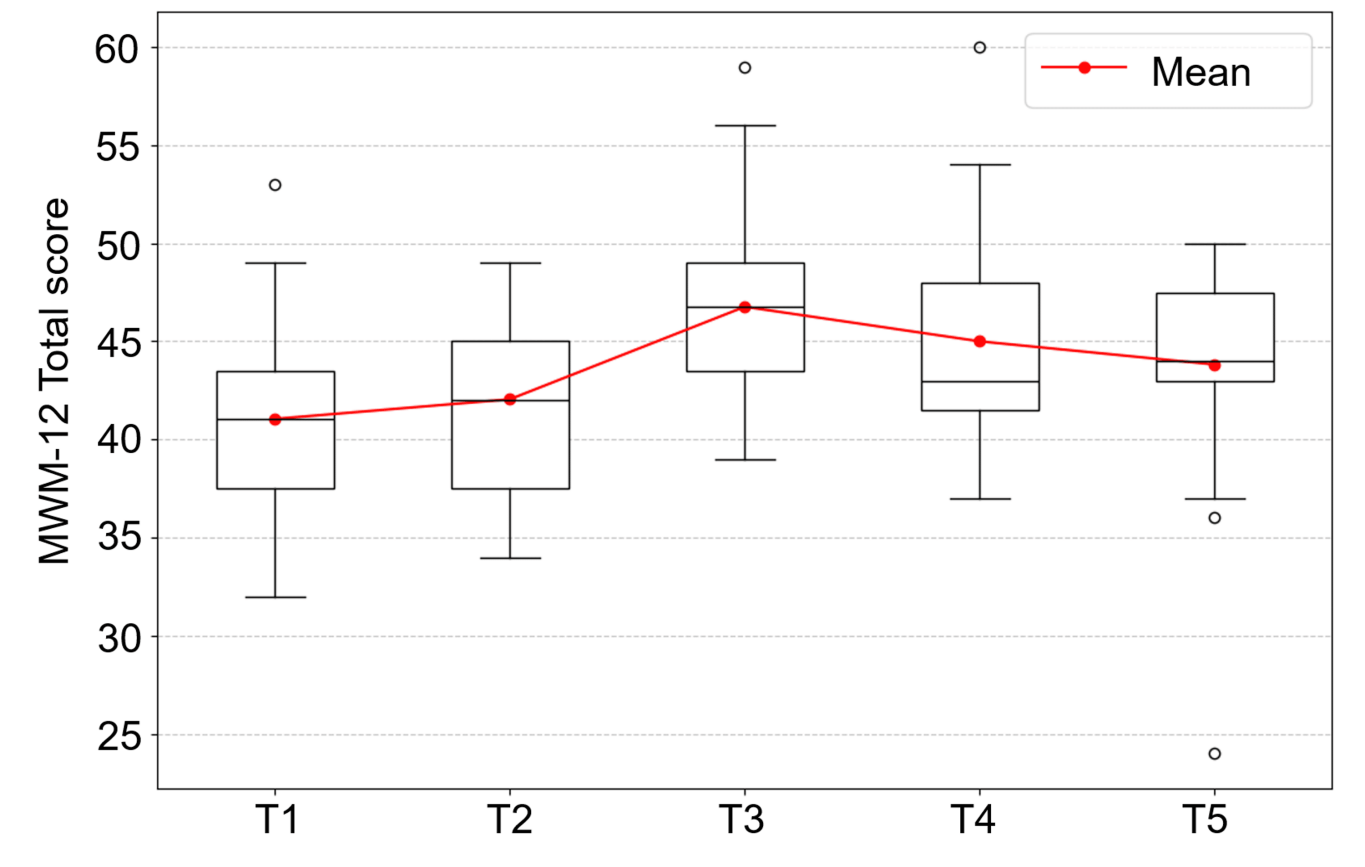
Longitudinal changes in MWM-12 total score (T1–T5) This figure displays the distribution of MWM-12 total scores at five time points (T1–T5) using box-and-whisker plots. The red line represents the mean MWM-12 total score across assessments. The mean ± SD at each time point was: T1, 41.0 ± 4.9; T2, 42.0 ± 4.6; T3, 46.8 ± 5.6; T4, 45.0 ± 5.7; and T5, 43.8 ± 5.7. MWM: Measure of Multifaceted Work Motivations

The number of responses at time points T1-T5 was 22, 22, 21, 22, and 22, respectively. The median (interquartile range: IQR) MWM-12 total score at each measurement point was as follows: T1, 41.5 (37.0 - 44.3); T2, 42.0 (37.0 - 45.5); T3, 47.0 (41.0 - 50.0); T4, 43.0 (41.0 - 48.0); and T5, 44.5 (43.0 - 48.0) (Figure 2). Corresponding mean ± SD were: T1, 41.0 ± 4.9; T2, 42.0 ± 4.6; T3, 46.8 ± 5.6; T4, 45.0 ± 5.7; and T5, 43.8 ± 5.7. A notable increase in the total MWM-12 score was observed between T2 and T3. Subsequently, the total scores and all 12 individual item scores of the MWM-12 were compared between T1 and T5.

The MWM-12 total score showed a statistically significant increase from T1 (median (IQR): 41.5 (37.0-44.3)) to T5 (44.5 (43.0-48.0); *Z*= -2.51,* p*= 0.01, *r*= 0.53). Significant increases were also observed for specific sub-items (Table 1), including the directionality of achievement-oriented motivation (M1: T1, 3 (3-4); T5, 4 (3-4); *Z*= -2.53, *p*= 0.01, *r*= 0.54), the directionality of competition-oriented motivation (M4: T1, 3 (2-3); T5, 3 (3-4); *Z*= -2.48, *p*= 0.01, *r*= 0.53), the sustainability of competition-oriented motivation (M6: T1, 2.5 (2-3); T5, 3 (2.8-4); *Z*= -2.21, *p*= 0.03, *r*= 0.47), and the sustainability of cooperation-oriented motivation (M9: T1,3 (3-4); T5 = 4 (3-4); *Z*= -2.12, *p*= 0.03, *r*= 0.45).

**Table 1:** Comparison of MWM-12 total scores and sub-scores at T1 and T5 Data presented as median (interquartile range, IQR). Changes in scores between T1 and T5 were analyzed using the Wilcoxon signed-rank test. *Z* represents the standardized test statistic derived from the Wilcoxon signed-rank test. *p* values are two-tailed. Significance thresholds: * *p* < 0.05. The effect size r was calculated as *Z/√n*. Effect sizes were interpreted as small (0.10), medium (0.30), and large (0.50) according to Cohen. CADP: Course for Academic Development of Psychiatrists; MWM: Measure of Multifaceted Work Motivations; IQR: interquartile range

		T1: Two weeks before the CADP (n=22)	T5: Three months after the CADP (n=22)	Z	p	r
		Median (IQR)	Median (IQR)			
MWM-12 total scores	Sum of M1-M12	41.5 (37.0-44.3)	44.5 (43.0-48.0)	-2.51	0.01*	0.53
Achievement-oriented motivation	M1: Directionality	3 (3-4)	4 (3-4)	-2.53	0.01*	0.54
	M2: Intensity	3.5 (3-4)	4 (3-4)	-1.16	0.25	0.25
	M3: Sustainability	3.5 (3-4)	4 (3-4)	-1.94	0.05	0.41
Competition-oriented motivation	M4: Directionality	3 (2-3)	3 (3-4)	-2.48	0.01*	0.53
	M5: Intensity	3 (2-4)	3 (2.8-4)	-0.58	0.56	0.12
	M6: Sustainability	2.5 (2-3)	3 (2.8-4)	-2.21	0.03*	0.47
Cooperation-oriented motivation	M7: Directionality	4 (3-4)	4 (3-4)	-0.28	0.78	0.06
	M8: Intensity	4 (3-4)	4 (3-4)	0.00	1.00	0.00
	M9: Sustainability	3 (3-4)	4 (3-4)	-2.12	0.03*	0.45
Learning-oriented motivation	M10: Directionality	4 (3-5)	4 (4-4.3)	-0.97	0.33	0.21
	M11: Intensity	4 (3-4)	4 (4-4)	-0.54	0.59	0.11
	M12: Sustainability	4 (3-4)	4 (3.8-4)	-0.94	0.35	0.20

In summary, within the directionality dimension of work motivation, significant improvements were observed in M1 and M4 motivations. Within the sustainability dimension, notable increases were noted in M6 and M9 motivations.

Qualitative analysis

Coding and Categorization

Qualitative analysis of the open-ended responses regarding participation in the CADP (hereinafter referred to as "the course") resulted in the classification of codes into six categories: C1: English skills; C2: Growth as a psychiatrist; C3: Personal networking; C4: Personal growth; C5: Increased motivation; and C6: Characteristics of the CADP. These categories were further subdivided into subcategories based on the content of responses from the pre- and post-course surveys. Table 2 summarizes the identified categories, representative subcategories, and illustrative excerpts from participants’ comments.

**Table 2:** Category and example of participants’ responses This table presents exemplary participant responses grouped by category and subcategory. Open-ended responses were collected via a pre-course survey at Time 2 (T2: immediately before CADP, March 3-7), focusing on "Expectations to attend CADP" and "Purposes to attend CADP." A subsequent post-course survey at Time 3 (T3: immediately after CADP, March 10-16) gathered insights on "Gains through CADP," "Findings through CADP," and "General impressions through CADP." Responses originating from the pre-course survey are indicated by (Pre), those from the post-course survey by (Post), and responses common to both by (Pre & Post). Participant identifiers (e.g., P1) denote the individual who provided the response. CADP: Course for Academic Development of Psychiatrists

Category	Subcategory	Example of participants' responses
C1. English skills	Improving English skills (Pre)	P17. “I want to improve English language skills”
	Motivation to learn English (Pre & Post)	P19. “Motivation to learn English”
	Personal experience about English (Post)	P14. “I was able to speak English better than last time.”
	No need for perfect English (Post)	P23. “It does not have to be perfect English.”
C2. Growth as a psychiatrist	Academic skills (Pre & Post)	P11. “I learned many specific techniques and points to make good oral and poster presentations.”
	Learning from experts and colleagues (Pre & Post)	P2. “All the participants had enthusiasm for psychiatric care, which was very inspiring.” P11. “I was exposed to Prof. Sartorius' wealth of knowledge and his very human and warm philosophy.”
	Changes in view of careers (Post)	P2. “It was a good opportunity for me to think about my future career path.” P8. “The lectures by CADP graduates and experts helped me to understand the success stories of our seniors and to have a better perspective on the future.”
C3. Personal networking	Interacting with people (Pre & Post)	P13. “I was able to interact with highly motivated psychiatrists.” P16. “I was able to interact with participants from various backgrounds.”
	Networking (Pre & Post)	P11. “I was able to spend dense time with many psychiatrists from Japan and abroad, forming bonds that will continue in the future.”
	Importance of network (Post)	P20. “I rediscovered the importance of human connections.”
C4. Personal growth	Inspired by others (Post)	P11. “I was greatly inspired by interacting with motivated early and mid-career psychiatrists.”
	Leadership (Post)	P10. After listening to Prof. Sartorius' lecture, I updated my view of leadership.
	Changes during the first participation (Post)	P2. “At first, I was very anxious and nervous because I was not confident in my English and did not know anyone, but through group work and the reception, I got to know more people and gradually started to enjoy myself.” P9. “I was extremely nervous on the first day, but through self-introductions, small group work, and the reception party, I became more comfortable interacting with other participants.”
	Changes through continued participation (Post)	P14. “I was able to enjoy the interaction with the participants because I had a little more mental capacity than the first time I participated.” P20. “I was most pleased to see that the participants, who looked stiff at first, gradually relaxed as the days went by, and I could see their smiles and their willingness to speak up.” P23. “This was my fourth time to participate in CADP. The first time I participated, I had no experience in writing English papers, but I found myself writing papers in English every year, earning a degree, and even studying abroad, which I think is a true effect of participating in CADP.”
C5. Increased motivation	Increased motivation (Post)	P12. “I got really motivated.”
	Increased confidence and challenging spirit (Post)	P5. “I would like to challenge myself in many things.” P11. “I felt that I will be able to be confident when I present at international conferences in the future. It has motivated me to improve my future research and clinical practice.” P16. “I thought that by trying more and more without fear of failure, I will discover new things. I would like to continue to broaden my horizons and gain more experience.”
C6. Characteristics of the CADP	Continuity of CADP (Pre & Post)	P21. “I want to pass on JYPO activities to the next generation.”
	Characteristics of the CADP (Post)	P1. “It was an opportunity to have an experience that I cannot have in my daily practice. There were lectures, presentations, and a lot of fun and satisfying content.” P13. “CADP is a three-day, two-night long event. I have an image of CADP as a place where excellent and enthusiastic doctors gather.” P14. “I found the entire meeting to be warm and friendly from start to finish. This kind of connection is hard to come by on other occasions and I felt it was a wonderful experience.”

Changes in the Trends of Open-Ended Responses Before and After the Course

Table 3 illustrates the frequency of each category’s appearance in participant responses. Prior to the course, Japanese participants commonly expressed expectations related to C1 and C3. After the course, reports of gains in C2, C3, C4, and C5 were more frequent. In addition, mentions of C6 appeared more frequently in post-course feedback.

**Table 3:** Number of occurrences of each category in participants' open-ended responses Open-ended responses regarding "Expectations to attend CADP" and "Purposes to attend CADP" were collected via a pre-course survey at Time 2 (T2: immediately before CADP, March 3-7). Subsequently, a post-course survey at Time 3 (T3: immediately after CADP, March 10-16) gathered responses concerning "Gains through CADP," "Findings through CADP," and "General impressions through CADP." For each participant's response to each open-ended question, the presence (1) or absence (0) of content belonging to a specific category was recorded. The table values represent the summation of these binary assignments across all participants for each category. CADP: Course for Academic Development of Psychiatrists

Time stage	T2 (n = 22)			T3 (n = 21)			
Question	Expectations to attend CADP	Purposes to attend CADP	Total mentions across all responses	Gains through CADP	Findings through CADP	General impressions through CADP	Total mentions across all responses
C1. English skills	7 (32%)	7 (32%)	9 (41%)	5 (24%)	5 (24%)	7 (33%)	12 (57%)
C2. Growth as a psychiatrist	5 (23%)	7 (32%)	9 (41%)	8 (38%)	7 (33%)	11 (52%)	16 (76%)
C3. Personal networking	14 (64%)	13 (59%)	18 (82%)	15 (71%)	5 (24%)	14 (67%)	18 (86%)
C4. Personal growth	0 (0%)	0 (0%)	0 (0%)	2 (10%)	7 (33%)	18 (86%)	19 (90%)
C5. Increased motivation	0 (0%)	0 (0%)	0 (0%)	4 (19%)	1 (5%)	5 (24%)	7 (33%)
C6. Characteristics of the CADP	0 (0%)	4 (18%)	4 (18%)	0 (0%)	4 (19%)	17 (81%)	17 (81%)

C1 (English skills): Prior to the course, participants expressed a desire to improve their English proficiency. During the course, they engaged in presentations and group work conducted in English. Following the course, numerous participants reported perceived improvements and expressed willingness to continue refining their English. Some participants also noted that complete fluency was not always necessary and that communicating in simple, clear English could often be more effective.

C2 (Growth as a psychiatrist): Before the course, participants expressed expectations for professional growth, particularly enhancing their presentation skills and broadening their perspectives as psychiatrists. During the course, they reported gaining experience in presenting and exchanging ideas, and learning techniques to effectively communicate with an audience. Some participants also noted that their interactions with lecturers, CADP alumni, and peers contributed to shifts in their career outlook.

C3 (Personal networking): Before the course, the participants reported their expectations of interacting with other attendees and establishing professional networks. During the course, they noted that they engaged with peers from diverse backgrounds, who were highly motivated in their work. Some participants expressed a desire to maintain the connections they established throughout their course.

C4 (Personal growth): This category was not mentioned during the pre-course but prominently emerged in post-course feedback. Participants indicated that interactions with peers from diverse backgrounds and with high work motivation served as sources of inspiration. Furthermore, several participants noted the acquisition of leadership skills that they regarded as essential to their professional development.

First-time attendees reported initial feelings of anxiety and nervousness, but gradually found enjoyment in the course through interactions with other participants and shared growth experiences. They also experienced a sense of accomplishment and growth by navigating challenges in an unfamiliar yet stimulating environment. Improved self-efficacy appeared to be linked to more positive engagement in learning experiences.

Repeat attendees reported greater relaxation and recognition of their personal growth compared to their initial participation. Those involved in organizing the event also reported enhanced learning in areas such as leadership and empathy. These experiences were attributed to JYPO’s structure as a youth-led organization, where CADP participants typically graduate after attending four sessions. Junior members were aware of inheriting the CADP legacy from their seniors, whereas senior members consciously sought to pass on their knowledge and experiences to the next generation.

Some participants reported significant career advancement owing to increased self-confidence and willingness to embrace new challenges fostered by the CADP experience.

C5 (Increased motivation): This category, distinct from personal growth, was not present in pre-course responses but appeared clearly in post-course feedback. Participants explicitly cited increased motivation, heightened self-confidence, and heightened willingness to tackle new challenges. These outcomes were attributed to their active engagement in communication and relationship-building skills within the academic setting of the course. Many felt that the experience empowered them to adopt a more proactive approach in their professional lives.

C6 (Characteristics of the CADP): In the post-course survey, participants frequently commented on the distinctive characteristics of the CADP. Several key features were highlighted, including its unique structure as a three-day, two-night residential training program; its international and diverse environment characterized by English as the official language; the presence of participants from various countries, regions, and professional backgrounds; and the supportive and welcoming attitudes of the program’s organizers, which contributed significantly to their overall experience.

Summary of the Participants’ Responses Through the Course

Participants’ responses before and after the course are summarized in Table 4.

**Table 4:** Summary of the participants’ responses through their participation in CADP Qualitative responses from participants were categorized into C1 to C6. The "Before the Course" column summarizes initial expectations and purposes for attending CADP, while "After the Course" reflects gains, key findings, and general impressions from CADP. CADP: Course for Academic Development of Psychiatrists

Category	Before the course	After the course
English skills (C1)	To improve English skills, especially presentation skills.	Improved English presentation skills; Realized that perfect English was not necessary.
Growth as a psychiatrist (C2)	To gain experience and broaden perspectives.	Improved academic skills; Learning from experts and peers; Changed views about their career.
Personal networking (C3)	To build new human networks through interaction with other psychiatrists.	Built lasting relationships with diverse and motivated participants.
Personal growth (C4)	(Not specifically stated before the course.)	First-time participants gradually adjusted and felt a sense of personal growth; Learned leadership; Inspired by diverse participants.
Increased motivation (C5)	(Not specifically stated before the course.)	Motivated to improve clinical and research skills; Learned to face challenges without fear of failure.
Characteristics of the CADP (C6)	Multiple-time participants seek to cultivate junior successors in workshop management and participation.	International setting; English official language; Activities included presentations, group work, and lectures; Warm and friendly atmosphere; Managed by early-career psychiatrists.

Process of changes in work motivation

Integrating the quantitative and qualitative results enabled an examination of the processes underlying changes in work motivation during the course. The following factors were identified as likely contributors to these changes. Personal growth experienced through participation in the CADP was associated with an overall increase in work motivation. Shifts in career perspectives and the development of a clearer career vision were linked to higher scores on the item reflecting career direction (M1). Improved English proficiency, professional development, and interactions with motivated peers and mentors during CADP may increase motivation for high-level performance (M4). Enhanced self-confidence and willingness to embrace challenges, fostered by personal growth, mutual support, and networking with highly motivated colleagues, likely reinforced sustained motivation for goal achievement (M6) and collaboration with colleagues (M9).

## Discussion

Understanding the process of change

The integration of quantitative and qualitative analyses enabled an in-depth examination of the processes underlying changes in work motivation throughout the course.

Changes in the directionality of achievement-oriented motivation (M1)

Strengthening professional identity as a physician is widely recognized as a key factor in enhancing resilience against burnout and cynicism [13] and likely contributes to sustaining high levels of work motivation. Lectures delivered by leading experts and CADP graduates, along with their shared career experiences, played a crucial role in fostering professional identity, offering role models, and shaping participants’ career perspectives. These experiences may explain the observed increase in scores related to career direction (M1). In addition, interactions with other enthusiastic participants appeared to positively influence career outlook. Notably, the philosophical insights special lecturer at the CADP were reported as deeply inspiring by some participants.

Changes in the directionality of competition-oriented motivation (M4)

The observed increase in motivation to perform at a higher level (M4) may be attributed to several factors. First, participants reported improvements in their academic skills and personal growth through lectures and practical group work. Given that Japanese medical students commonly report low English proficiency and confidence, coupled with limited opportunities for practice, particularly in academic presentations [26], many participants noted improvements in their English skills. Second, developing a personal network of enthusiastic peers enables friendly competition. Third, interactions with colleagues from diverse cultural backgrounds promoted appreciation of diversity, which is an essential competency for psychiatrists. Importantly, this exposure to diverse perspectives may also encourage participants to engage in reflective self-relativization, a process that has been shown to contribute to professional identity formation [27].

Changes in the sustainability of competition-oriented motivation (M6)

Previous studies on healthcare professionals have shown that self-efficacy and self-esteem are significant sources of intrinsic motivation, suggesting a correlation between positive self-evaluation and higher levels of work motivation [28]. The observed increase in motivation to continue working until the results were achieved (M6) may be attributed to enhanced self-confidence and greater willingness to embrace challenges, which were fostered through personal and professional growth in activities that necessitated active participation, such as presentations and practical group work.

Changes in the sustainability of cooperation-oriented motivation (M9)

One advantage of shared leadership training is the opportunity to engage with and learn from individuals across institutions. This emphasizes the value of cross-institutional interactions in stimulating residents and enhancing motivation [29]. The intensive, immersive nature of CADP likely facilitated the development of relationships among the participants. The observed increase in motivation to sustain collaboration with colleagues (M9) may be attributed to growth through mutual evaluation during presentations and practical group work requiring peer cooperation, as well as shared time and environment throughout the course.

Other MWM-12 items with little or no change

In contrast, certain elements of work motivation exhibited minimal or no change, potentially for the following reasons. CADP participants applied voluntarily, were fully aware that the program was a three-day, two-night residential training conducted in English, and were composed of presentation-based and group-work-oriented sessions that required active participation. Consequently, participants were likely to possess high initial motivation to learn, even before the course commenced. This may explain why all three dimensions of learning-oriented motivation (M10-12) were already high prior to the course and did not exhibit substantial changes.

Similarly, applicants demanding active engagement with their peers are expected to have a cooperative mindset before attending. This may account for the high pre-course scores in both the directionality and intensity of cooperation-oriented motivation (M7-8), which likewise showed minimal changes after the course.

CADP as a leadership development training

Leadership development training programs should be designed systematically to enhance leaders’ knowledge, skills, and competencies. Generally, such training aims to strengthen overall organizational capabilities. These programs adopt an integrated approach that emphasizes not only the interaction between leaders and followers but also broader social and contextual factors. Leadership development encompasses both individual-level growth and systemic perspectives. A meta-analysis examining the effectiveness of leadership development programs identified several key features of highly effective training [30]. These features include having clear objectives aligned with participants’ needs, incorporating appropriate training methods such as hands-on practice and demonstrations, providing continuous feedback to clarify areas for improvement, and including a diverse range of content from interpersonal skills to project development. Effective training also tends to use face-to-face delivery and elicit positive reactions from trainees, which enhances their motivation to learn.

CADP embodies many of the characteristics of effective leadership development training. It offers a systematic curriculum, facilitates international exchange, and provides opportunities to learn from experts and CADP alumni, while enabling the acquisition of practical academic and leadership skills. Notably, the program was specifically designed to foster the development of early-career psychiatrists and to equip them with the competencies necessary for success in international academic environments. CADP is planned and managed by Steering Committee members who are early-career psychiatrists, thereby ensuring that the program reflects the specific needs of its target audience. This peer-driven management structure likely contributed to the highly positive evaluations reported by participants. Furthermore, CADP provides a platform for early-career psychiatrists to exchange feedback and cultivate both personal and professional growth within a supportive, network-based learning environment.

Limitations and solutions of the online longitudinal survey

This study has some limitations that warrant consideration. First, the limited sample size, consisting solely of Japanese participants from a single CADP cohort, restricts the generalizability of the findings. Future studies should incorporate larger and more diverse samples to improve the robustness and external validity of the results.

Second, this study employed a within-subject design with five repeated measurements, using the first two as a baseline to evaluate changes following the intervention. This approach allowed each participant to serve as their own control, enabling a more precise evaluation of changes brought about by the intervention. However, the absence of a separate control group makes it difficult to　completely rule out the possibility that the observed changes were influenced by external factors, such as natural changes over time or the placebo effect.

Third, reliance on self-reported data collected through an online longitudinal survey introduced potential biases over time, including recall, social desirability, and attrition biases. Future studies may benefit from adopting a mixed-methods approach, combining self-reported surveys with periodic in-person interviews or real-time data collection. Such an approach could enhance data reliability by capturing immediate and detailed responses, thereby mitigating recall bias.

Fourth, the exclusive use of English as the official language of the CADP may have affected participants heterogeneously based on their English proficiency. Individuals with lower proficiency may have encountered difficulties fully engaging in discussions and group work, potentially influencing their learning outcomes. Future research should consider assessing English proficiency and its influence on motivation and exploring support strategies such as preparatory language training or mentorship. However, it is also important to acknowledge that, despite the initial challenges, many participants reported reduced apprehension toward using English and increased motivation to improve their language skills as a result of their participation. This finding suggests that CADP may play a positive role in fostering English communication skills and confidence, which is a meaningful outcome.

Finally, implementing measures to ensure participant anonymity, along with consistent reminders, may help increase response rates and minimize dropouts, thereby improving the validity and continuity of the longitudinal findings. These strategies could contribute to a more comprehensive understanding of the long-term impact of educational programs, such as CADP, on the motivation and career development of early-career psychiatrists.

## Conclusions

Peer-led educational courses such as the CADP appear to positively impact work motivation among early-career psychiatrists. Participants in the 21st CADP reported improvements in their perspectives on professional skill development, international networking, personal growth, and long-term career planning. These factors likely contributed to enhanced confidence and a greater willingness to embrace new challenges, thereby augmenting work motivation. Incorporating these elements into future courses may effectively support the professional development and motivation of early-career psychiatrists.

## Appendices

Appendix 1　

*Details About the Course for Academic Development of Psychiatrists* (*CADP) and Related Courses*

The CADP features diverse sessions, including oral and poster presentations, special lectures, small group work, and social gatherings. The course was designed to maximize participant involvement, providing opportunities for individual presentations, comment sessions during peer presentations, and continuous involvement in group work and other activities. As a result of active participant engagement, it was not uncommon for vigorous discussions and collaborative efforts concerning group work presentations to extend beyond scheduled program hours.

Participants are assigned roles such as oral and poster presenters, and chairpersons, based on their cumulative participation. These evolving roles foster new discoveries with each attendance. Within the CADP Steering Committee, a structured system ensures that participants, after their initial involvement, serve as steering committee member, eventually graduating after their fourth participation.

A distinctive feature, uniquely cultivated by CADP/JYPO (Japan Young Psychiatrists Organization) and unparalleled in similar training programs elsewhere, is the recurrent involvement of CADP graduates. CADP graduates can attend as advisors, simultaneously furthering their own learning and mentoring junior colleagues. A dedicated "My Career and CADP" session allows CADP graduates to discuss how the program has influenced their professional paths.

Appendix 2　

Codebook

The following C1-6 are categories of the pre- and post-course survey responses.

Each category has the following subcategories: examples of codes shown in ( / / ), definitions, and examples of the participant's response.

The icon below shows whether the subcategory appeared in the pre- and/or post-course survey.

*(Pre)　 Appeared only in the pre-course survey.

*(Post)　 Appeared only in the post-course survey.

*(Pre & Post)　 Appeared in both pre- and post-course surveys.

C1. English Skills

Participants' responses related to English skills.

Subcategories (Example of Codes):

- Improving English skills (Improving English Skills / Improving English (Discussion / Presentation / Speaking) Skills / Improving English Skills to manage training course) *(Pre)

    Responses regarding improving English skills.

        P17.  I want to improve English language skills

- Motivation to learn English (Motivation to learn English) *(Pre & Post)

    Responses regarding motivation for learning English.

        P19.  Motivation to learn English

- Personal Experience about English (Improvement of English Communication / Personal experience about English Communication) *(Post)

    Responses regarding personal experiences with English communication during the course.

        P14.  I was able to speak English better than last time.

- No Need for Perfect English (No Need for Perfect English) *(Post)

    Responses mentioning that perfect English is not necessary.

        P23.  It does not have to be perfect English.

C2. Growth as a Psychiatrist

Participants' responses related to professional growth as a psychiatrist, including knowledge acquisition, academic skills, and broadening perspectives.

Subcategories (Example of Codes):

- Academic Skills (Chairmanship Skill / Presentation Skill / Project Planning Skill / Research Skill ) *(Pre & Post)

    Responses regarding academic skills, such as skills for presentation, chairmanship, research and project planning.

        P8.  Improvement of presentation skills

- Broad perspective (Broad perspective) *(Pre & Post)

    Responses related to broadening perspectives.

        P6.  To get my view wider

- Knowledge (Learning about psychiatry) *(Pre & Post)

    Responses related to knowledge acquisition.

        P3.  Learning about psychiatry

- Learning from experts and colleagues (Learning from colleagues / Learning from others/ Learning from experts/ lectures/ graduates ) *(Pre & Post)

    Responses regarding learning from others: colleagues, experts, lectures and graduates.

        P13.  I want to know what psychiatrists are interested in.

- Changes in view of careers (Career development / Future Career / Future Perspectives )

    Responses regarding career development and future perspectives. *(Post)

        P2.  It was a good opportunity for me to think about my future career path.

C3. Personal Networking

Participants' responses related to interacting, building relationships, networking with colleagues, and strengthening professional connections.

Subcategories (Example of Codes):

- Interacting with People (Interacting with People / Interacting with People from oversea / Interacting with People (domestic and oversea) ) *(Pre & Post)

    Responses related to interacting but did not specifically mention building relationships, networking with colleagues, or strengthening professional connections.

        P5.  I would like to join with psychiatrists across the country.

- Networking (Networking) *(Pre & Post)

    Responses regarding building relationships, networking with colleagues, and strengthening professional connections.

        P20.  Continuous connection

- Importance of Network (Importance of Network) *(Post)

     Responses mentioning they had learned or understood the importance of human networks.

         P20.  I rediscovered the importance of human connections.

C4. Personal Growth

Participants' responses related to personal development, including leadership, and self-awareness changes.

Subcategories (Example of Codes):

- Inspired by others (Building Good Relationships / Friendly competition / Inspired by others / Trial and Error with others) *(Post)

    Responses related to being inspired through the interaction with others.

        P11.  I was greatly inspired by interacting with motivated young and mid-career psychiatrists.

- Leadership (Learning about Leadership / Leadership Experience) *(Post)

    Participants' responses related to leadership.

        P10.  After listening to Prof. Sartorius' lecture, I updated my view of leadership.

- Personal experience (Personal experience) *(Post)

    Participants' responses related to personal experiences.

        P17.  Although I was not in perfect physical condition, I was able to give my best effort.

- Changes during the first participation (Changes during the first participation) *(Post)

    Participants' responses related to the changes during the first participation.

        P2.  At first, I was very anxious and nervous because I was not confident in my English and did not know anyone, but through group work and the reception, I got to know more people and gradually started to enjoy myself.

- Changes through continued participation (Changes through continued participation) *(Post)

    Participants' responses related to the changes through continued participation.

        P14.  I was able to enjoy the interaction with the participants because I had a little more mental capacity than the first time I participated.

C5. Increased Motivation

Participants' responses related to motivation changes.

Subcategories (Example of Codes):

- Increased motivation (Increased motivation) *(Post)

    Participants' responses related to motivation changes.

        P12.  I got really motivated.

- Increased confidence and challenging spirit (Challenging spirits / Decreased Fear of Changes / Increased confidence) *(Post)

    Participants' responses related to increased confidence and challenging spirit.

        P16.  I thought that by trying more and more without fear of failure, I will discover new things. I would like to continue to broaden my horizons and gain more experience.

C6. Characteristics of the CADP

Participants' responses related to characteristics of the CADP program, including its impact and continuity.

Subcategories (Example of Codes):

- Continuity of CADP (Continuity of CADP/ The time for Graduation / Trans-generational succession / JYPO as a basis for succession) *(Pre & Post)

    Participants' responses related to the continuity of the CADP.

        P21.  I want to pass on JYPO activities to the next generation.

- Development of JYPO (Development of CADP) *(Pre)

    Participants' responses related to the development of JYPO.

        P14.  To increase the number of JYPO members

- Developments in the mental health field (Development in the mental health field) *(Pre)

    Participants' responses related to development in the mental health field.

        P15.  I hope to help make the mental health community more lively

- Characteristics of the CADP (Appreciation / Changes through multiple participation / Characteristics of participants / Characteristics of the CADP / Diversity / Experience in CADP / Growth within the meeting / Pleasant experience / Suggestions for improvement of CADP) *(Post)

    Participants' responses related to characteristics of the CADP.

        P1.  It was an opportunity to have an experience that I cannot have in my daily practice. There were lectures, presentations, and a lot of fun and satisfying content.

Appendix 3

**Table 5:** Participants’ profiles Japanese participants included one junior resident (pre-psychiatric specialization) and two medical students, all classified into the '3 years or less' group in terms of psychiatric experience. JYPO: Japan Young Psychiatrists Organization; CADP: Course for Academic Development of Psychiatrists

No.	Nationality	Sex	Number of CADP participation	Psychiatric experience (3 years or less, 4 to 6 years, 7 years or more)	JYPO member	Role in CADP
P1	Japan	M	1	3 years or less	No	Others
P2	Japan	M	1	3 years or less	No	Others
P3	Japan	F	1	3 years or less	No	Others
P4	Japan	M	1	3 years or less	No	Others
P5	Japan	M	1	3 years or less	Yes	Others
P6	Japan	M	1	3 years or less	Yes	Others
P7	Japan	F	1	3 years or less	Yes	Others
P8	Japan	M	1	3 years or less	Yes	Others
P9	Japan	M	1	4 to 6 years	Yes	Others
P10	Japan	M	1	4 to 6 years	Yes	Others
P11	Japan	M	1	7 years or more	No	Others
P12	Japan	M	2	3 years or less	No	Others
P13	Japan	M	2	3 years or less	Yes	Others
P14	Japan	M	2	3 years or less	Yes	Steering Committee Member
P15	Japan	M	2	4 to 6 years	Yes	Steering Committee Member
P16	Japan	M	2	4 to 6 years	Yes	Steering Committee Member
P17	Japan	M	2	4 to 6 years	Yes	Steering Committee Member
P18	Japan	M	2	7 years or more	Yes	Others
P19	Japan	M	3	4 to 6 years	Yes	Others
P20	Japan	F	3	4 to 6 years	Yes	Steering Committee Member
P21	Japan	M	3	7 years or more	Yes	Steering Committee Member
P22	Japan	M	4	7 years or more	Yes	Steering Committee Member
P23	Japan	F	4	7 years or more	Yes	Steering Committee Member
P24	Asian country	M	1	–	–	–
P25	Asian country	F	1	–	–	–
P26	Asian country	M	1	–	–	–
P27	Asian country	F	1	–	–	–
P28	Asian country	M	1	–	-–	–

## References

[1] Mitchell TR Matching motivational strategies with organizational contexts. Research in Organizational Behavior.

[2] Veenstra GL, Dabekaussen KF, Molleman E, Heineman E, Welker GA (2022). Health care professionals' motivation, their behaviors, and the quality of hospital care: a mixed-methods systematic review. Health Care Manage Rev.

[3] Bodenheimer T, Sinsky C (2014). From triple to quadruple aim: care of the patient requires care of the provider. Ann Fam Med.

[4] Lagarde M, Huicho L, Papanicolas I (2019). Motivating provision of high quality care: it is not all about the money. BMJ.

[5] Koreki A, Nakagawa A, Abe A (2015). Mental health of Japanese psychiatrists: the relationship among level of occupational stress, satisfaction and depressive symptoms. BMC Res Notes.

[6] de Oliveira GS Jr, Chang R, Fitzgerald PC, Almeida MD, Castro-Alves LS, Ahmad S, McCarthy RJ (2013). The prevalence of burnout and depression and their association with adherence to safety and practice standards: a survey of United States anesthesiology trainees. Anesth Analg.

[7] Kumar S (2007). Burnout in psychiatrists. World Psychiatry.

[8] Umene-Nakano W, Kato TA, Kikuchi S, Tateno M, Fujisawa D, Hoshuyama T, Nakamura J (2013). Nationwide survey of work environment, work-life balance and burnout among psychiatrists in Japan. PLoS One.

[9] Tateno M, Kato TA, Uehara-Aoyama K (2017). The International Study of Burnout Syndrome Among Psychiatric Trainees (BoSS International): findings from Statistical Analysis of the Japanese Data (BoSS Japan) (Article in Japanese). Seishin Shinkeigaku Zasshi.

[10] Ryan RM, Deci EL (2000). Self-determination theory and the facilitation of intrinsic motivation, social development, and well-being. Am Psychol.

[11] Afolabi A, Fernando S, Bottiglieri T (2018). The effect of organisational factors in motivating healthcare employees: a systematic review. Br J Health Care Manag.

[12] Weldegebriel Z, Ejigu Y, Weldegebreal F, Woldie M (2016). Motivation of health workers and associated factors in public hospitals of West Amhara, Northwest Ethiopia. Patient Prefer Adherence.

[13] Wald HS (2015). Professional identity (trans)formation in medical education: reflection, relationship, resilience. Acad Med.

[14] Cornett M, Palermo C, Ash S (2023). Professional identity research in the health professions-a scoping review. Adv Health Sci Educ Theory Pract.

[15] Bhugra D (2015). Excellence in training - what makes a good psychiatrist?. Eur Psychiatry.

[16] Sartorius N (2013). Acquiring leadership skills: description of an international programme for early career psychiatrists. Leadership in Psychiatry.

[17] Krupchanka D, Pinto da Costa M, Jovanović N (2019). Norman Sartorius: psychiatry's living legend. Lancet Psychiatry.

[18] Tateno M (2011). 10th anniversary of the training course for Japanese early career psychiatrists: Course for the Academic Development of Psychiatrists (CADP): 10th anniversary of CAPD. Asia-Pac Psychiatry.

[19] Rai Y, Karki U, Thapaliya S, Bhattarai D, Hein S, Sanchez V (2020). Educational opportunities for Nepalese early career psychiatrists and trainees. J Psychiatr Assoc Nepal.

[20] Satake Y, Kuramochi I, Kawagishi R, Masuda M, Aki M, Oya N (2024). The pros and cons of virtual networking events: online exploratory survey of psychiatrists' opinions. BJPsych Int.

[21] Kawagishi R, Kuramochi I, Satake Y (2023). Report on the 1st and 2nd Japan Young Psychiatrists Organization online international networking meetings (JOIN meetings) in 2021 and 2022. Asian J Psychiatr.

[22] Uehara K, Tateno M, Takahashi H, Sugiura K (2007). Experiences and Insights from the World Congress of Psychiatry in Istanbul (Article in Japanese). Seishin Shinkeigaku Zasshi (Psychiatria et Neurologia Japonica).

[23] Takahashi H (2025). A perspective on the activities of the Japanese Society of Psychiatry and Neurology through the Japan Young Psychiatrists Organization (Article in Japanese). Seishin Shinkeigaku Zasshi (Psychiatria et Neurologia Japonica).

[24] Ikeda H, Morinaga Y (2017). Developing a measure of multi-faceted work motivations in Japanese organizations (Article in Japanese). Jpn Assoc Ind Organ Psychol J.

[25] Tong A, Sainsbury P, Craig J (2007). Consolidated criteria for reporting qualitative research (COREQ): a 32-item checklist for interviews and focus groups. Int J Qual Health Care.

[26] AlNouri M, Maniwa K, Asari T, Ishibashi Y (2023). Proposing a questionnaire for assessing English proficiency among Japanese medical students: current perspectives and a pilot survey. Cureus.

[27] Hayashi M, Son D, Nanishi K, Eto M (2020). Long-term contribution of international electives for medical students to professional identity formation: a qualitative study. BMJ Open.

[28] Kitsios F, Kamariotou M (2021). Job satisfaction behind motivation: an empirical study in public health workers. Heliyon.

[29] Levy KL, Sheffield V, Sturza J, Heidemann LA (2023). Important leadership skills and benefits of shared leadership training for chief residents: a Delphi analysis. J Healthc Leadersh.

[30] Lacerenza CN, Reyes DL, Marlow SL, Joseph DL, Salas E (2017). Leadership training design, delivery, and implementation: a meta-analysis. J Appl Psychol.

